# Effect of a Place-Based Learning Community on Belonging, Persistence, and Equity Gaps for First-Year STEM Students

**DOI:** 10.1007/s10755-020-09519-5

**Published:** 2020-07-24

**Authors:** Matthew D. Johnson, Amy E. Sprowles, Katlin R. Goldenberg, Steven T. Margell, Lisa Castellino

**Affiliations:** 1 Department of Wildlife, Humboldt State University, Arcata, CA USA; 2 Department of Biological Sciences, Humboldt State University, Arcata, CA USA; 3 Sponsored Programs Foundation, Humboldt State University, Arcata, CA USA; 4 Office of Institutional Effectiveness, Humboldt State University, Arcata, CA USA

**Keywords:** Opportunity gap, Retention, Quasi-experimental design, Hispanic, Indigenous

## Abstract

We combined tenets of learning communities and place-based learning to develop an innovative first-year program for STEM students. Using a quasi-experimental design, we found that participants in the place-based learning community had a stronger sense of belonging, improved academic performance, and increased first-year persistence relative to a matched reference group. We also showed that participation narrowed equity gaps in first-year outcomes for students underrepresented in the sciences. A sense of place arises not just from a location, but from interrelationships between people and the natural world, and these results suggest organizing learning around place can promote inclusive student success.

## Introduction

American higher education is fraught with inequity. College completion rates in the US continue to be tied disproportionately to race and ethnicity, socioeconomic status, and family history in higher education. There is a 20% gap in the rate of Black and Latinx students earning credentials compared to White and Asian students (Shapiro et al., 2017), and these gaps are even larger in science, technology, engineering, and math fields (hereafter, STEM; de Brey et al., 2019). Of the minoritized students who earn a bachelor of science degree, the percentage that continue on to complete a graduate degree is significantly lower than their majority counterparts (NCSES, 2015). Disproportional degree attainment creates a less diverse professional STEM workforce and small numbers of minoritized people in leadership positions, perpetuating the societal inequities that cause these issues (Gordon, 1986; Gándara, 1999). Closing these equity gaps is not only essential for improving science by bringing in a wider range of voices and viewpoints, it is a moral imperative (Hale, 2004; National Academy of Sciences, 2011; Riggs, 2018). While many institutions and national funders have launched special programs to support and train small numbers of top-performing STEM students from underrepresented groups (Hurtado, Cabrera, Lin, Arellano, & Espinosa, 2009), these activities are not sufficient to reverse the momentum of disparity (Asai, 2016). Broader institutional reforms are also urgently needed to disrupt the status quo and fundamentally alter how campuses welcome and support all STEM students (Graham, Frederick, Byars-Winston, Hunter, & Handelsman, 2013; Mack, Winter, & Soto, 2019; Olson & Riordan, 2012).

The first two years of college have been shown to be the most critical for recruiting and retaining STEM students (Olson & Riordan, 2012), but American colleges struggle to retain students in their first year (Clark, 2005; Burgette & Magun-Jackson, 2008). First-year student “learning communities” have emerged as a practice to improve student outcomes and narrow equity gaps between groups of students (Kuh, 2008; Otto, Evins, Boyer-Pennington, & Brinthaupt, 2015). Learning communities include “a variety of curricular approaches that intentionally link or cluster two or more courses, often around an interdisciplinary theme or problem, and enroll a common cohort of students” (Smith, MacGregor, Matthews, & Gabelnick, 2009, p. 20). Learning communities have the potential to improve student outcomes through several mechanisms (Weiss, Visher, Weissman, & Wathington, 2015). Strong peer-to-peer relationships are built when students are co-enrolled into two or more courses (Smith et al., 2009). If the course content is linked, students better understand connections and relationships among disciplines (Klein, 2005). When the community is designed to connect social and student support programs to the curriculum, relationships between faculty and students are strengthened and participants experience a greater level of engagement with campus life and academic identity (Tinto, 2003). Such psychosocial factors have been shown to be important for first year STEM students and linked to improved outcomes such as first year retention and graduation rates (Carrino & Gerace, 2016; Gillespie & Petitubin, 2016; Solanki, McPartlan, Xu, & Sato, 2019).

It is hypothesized that campuses with largely residential and full-time student populations may benefit most from the development of learning communities fostering a sense of belonging, because these are cases in which students must connect to a new community away from their familial home (Weiss et al., 2015). However, critics have noted that unless campuses can balance student integration with maintaining connections to families and communities back home, learning communities may be inappropriate for students of some cultures and/or for campuses with large commuter populations (Cabrera, Nora, Terenzini, Pascarella, & Hagedorn, 1999; González, 2002; Guiffrida, 2006). By sense of belonging, we mean “[the] perceived social support on campus, a feeling or sensation of connectedness, and the experience of mattering or feeling cared about, accepted, respected, valued by, and important to the campus community…” (Strayhorn, 2018, p. 4). Belonging is cultivated by connections, mutual respect of positionality, and recognition of the contribution of each person’s unique history to the new student community (O'Keeffe, 2013). For students from minoritized populations, it is imperative that institutions send an authentic message of welcoming and actively avoid campus cultures that suggest a need for students to assimilate (Hurtado et al., 2008). Indeed, the greatest barrier to a sense of belonging is a perceived need to “fit in” (Brown, 2017; Rendón, 1994).

Cultivating social and academic integration to a new campus home while honoring connections to familial homes is especially challenging and important for campuses that attract students of color to locales unlike that of their families (Garcia, 2019; Museus, 2014; Rendón, Garcia, & Person, 2004). One approach posited to balance these simultaneous needs is “place-based education.” A term more commonly referenced in the pre-collegiate and outdoor education pedagogical literature, place-based education seeks to connect students to the region of study, provide cultural and/or geographic context to lessons, and usually involves outdoor education methodologies (Gruenewald & Smith, 2014). Though the concept has recently been formalized, forward-thinking educators have promoted its ideas for over a century. In *School and Society*, John Dewey advocated learning in the local environment:

“Experience has its geographical aspect, its artistic and its literary, its scientific and its historical sides. All studies arise from aspects of the one earth and the one life lived upon it” (1915, p. 91, cited in Woodhouse & Knapp, 2000).

The approach seeks to draw lessons from a local lens to broader contexts and applications, generalizing to and contrasting with other regions, human communities, and ecosystems (Knapp, 2005; Semken & Freeman, 2008; Smith, 2002). It is related to Orr’s call to rethink education to build “ecoliteracy” so that students can understand the effects of knowledge on real people and their communities (Orr, 2004). However, more than simply contextualizing disciplines in a geography, a socially just placed-based education must give credence to the epistemological traditions that curate individual understandings of and relations to the social world (i.e., the place; Seawright, 2014). Moreover, to authentically engage students in place, the reality of systemically imposed oppression and violence must be understood and incorporated into curricula and lessons aimed at envisioning solutions to a place’s complex social and environmental challenges (Medina, 2013; Seawright, 2014). Destination campuses can foster a burgeoning sense of place in students without compromising their familial roots (Holton, 2015) by recognizing that “location itself is not enough to create a sense of place. It emerges from involvement between people, and between people and place” (Pretty, Chipuer, & Bramston, 2003, p. 274). In this way, place-based education provides a potential mechanism for students in unfamiliar settings to not only gain more regional familiarity, but also to better recognize unique aspects and parallels between communities near their campus and their familial homes (Kerby, 2015).

Over the last twenty years, much research has suggested that learning communities have a positive effect on student outcomes for students most at risk of not graduating (Brownell & Swaner, 2009; Otto et al., 2015; Weiss et al., 2015; Zhao & Kuh, 2004), including improving their persistence in STEM (Graham et al., 2013; Dagley, Georgiopoulos, Reece, & Young, 2016; Solanki et al., 2019). However, precious little of this research involves well-controlled experimental trials. The most rigorous analyses indicate statistically significant, though often modest, improvements in the academic performance of learning community participants (Sommo, Mayer, Rudd, & Cullinan, 2012; Weiss et al., 2015), with substantial variation among campuses in the magnitude of these positive effects (Otto et al., 2015). While several studies have suggested the benefits of learning communities (e.g., student engagement, retention, and academic achievement (e.g., Goldberg & Finkelstein, 2002; Stassen, 2003; Wilmer, 2009), a common limitation among them is the lack of an appropriate reference group. Because students often self-select into learning communities, they are unlikely representative of all non-participants, making raw comparisons vulnerable to self-selection bias and clouding interpretation of effect. True randomized control trials have been used to avoid such problems (as described in Sommo et al., 2012), but they are difficult to implement. Alternatively, a quasi-experimental design that employs analytical methods such as propensity score matching (Austin, 2011a) can statistically control for a set of measured background characteristics to create a reference set of students that better matches the student population in learning community.

In this study, we report findings from an innovative place-based learning community for first-year STEM students at a residential campus in Northern California. Our model is rooted in the broader theory that college persistence arises from a complex interaction of psychosocial, academic, and environmental factors, drawing on a combination of Tinto’s Student Integration Model (Tinto, 1975) and its revisions (e.g., Davidson & Wilson, 2013) with Bean’s Student Attrition Model (Bean, 1980; Bean & Metzner, 1985), merged into an integrated theory of persistence (Cabrera, Nora, & Castaneda, 1993; Kerby, 2015). The curricular structure was designed to improve motivation and academic achievement of participants from minoritized groups by linking the people, science, culture, values, and social justice issues of our local Indigenous community with the curriculum (Thoman, Brown, Mason, Harmsen, & Smith, 2014; Estrada et al., 2016; McGee & Bentley, 2017). This approach is aligned with national calls for integrating civic knowledge and engagement, as well as intercultural knowledge and competence, with academic content (National Leadership Council, 2007; American Chemical Society, 2010), a strategy demonstrated to improve motivation and academic achievement of participants from minoritized groups (Thoman et al., 2014; Estrada et al., 2016; McGee & Bentley, 2017).

This study examines the impact of participation in a place-based learning community on student persistence, equity gaps, and three of their broadly recognized predictors: students’ sense of social belonging, academic skills and attitudes, and academic performance in gateway courses. Below, we briefly describe the program’s setting, components, and student participants (more details are available in Johnson, Sprowles, Overeem, & Rich, 2017).

## Setting, Program Components, and Participants

This study of the influence of a place-based learning community on student outcomes was conducted at a mid-sized Master’s-granting state university located in northwestern California. The campus is located in a rural setting with a predominantly non-Hispanic White population (~75%, U.S. Census Bureau, 2010). It is home to nine Native American Tribes and resides on Wiyot ancestral land. The majority of first-year students are full-time and residential, and most students come from large urban centers elsewhere in California (San Francisco Bay Area and Southern California), with only 6% of students from the local area. By 2014, nearly half of the incoming first-year class was from an underrepresented group – defined here as students self-reporting as being African American, Hispanic or Latinx, American Indian or Alaska Native, Native Hawaiian or Pacific Islander, or two or more of these. In 2015, over 55% of the campus’s first-time undergraduates were first-generation students, defined here as students self-reporting to be in the first generation of their family to go to college, and this proportion was even higher (70%) among underrepresented students.

We launched our first place-based learning community for STEM students in fall 2015. Called the *Klamath Connection,* it links practices shown to improve first-year college student performance to a major feature of our geographic location: The Klamath River. The Klamath River Basin extends from Southern Oregon to the mouth of the river in northern California, an area encompassing over 15,750 miles^2^. It is inhabited by 120,000 people, 13% of which are Native American. Multiple environmental and social justice issues are associated with the region, such as conflicts over water rights and natural resource conservation, and issues affecting a diverse group of communities that include four Native American tribal nations: the Yurok, Hoopa, Karuk and Klamath Tribes. The issues of the Klamath are complex, engaging, and conspicuously multidisciplinary, providing a rich and nuanced context in which to explore interconnectedness of disciplines. The program involves students, faculty – many who have experience and expertise in the region – as well as staff and off-campus community partners including professional scientists, local Native American tribal members, and environmental restoration groups. Through integrated curriculum and activities, the program offers a substantively re-imagined first year experience for first-year STEM students (Johnson et al., 2017; Sprowles et al., 2019). To our knowledge it is one of the only attempts in the U.S. to make the learning community place-based by embedding an interdisciplinary focus on the landscape, people, and cultures of the University’s location. The programmatic structure of the Klamath Connection place-based learning community was designed to include four best practices for supporting first-year college students: a summer immersion, peer mentoring, a first-year seminar, and a cohort blocked scheduling of lower division courses required for their chosen STEM major.

### Summer Immersion

The summer immersion was a four day program comprised of activities designed to (1) welcome first-year students to the exciting and diverse place and the academic community of learners, (2) foster their scientific identity through the participation in hands-on, inquiry based activities with other scientists (3) introduce the outdoors as a “classroom,” (4) help students recognize that solving complex social and environmental problems requires interconnectedness of disciplines and working with others, (5) foster an appreciation for peer learning, and (6) introduce the campus community, offices and faculty, staff and students from academic and student affairs, that are here to support each student on their journey toward degree. To achieve these goals, the students arrived to campus four days before the standard freshman orientation. They were clustered by major so they could explore thematic content related to their academic year coursework with Klamath Connection peer mentors, staff and faculty as well as Native American tribal scientists, natural resource policy professionals, and cultural experts. Though the activities varied slightly over the three cohorts, all included a film about social and environmental challenges for the Klamath River and its communities, a shorter field trip to natural areas near campus, and a visit to the reservation of the Yurok Tribe. Students also participated in two academic components designed to familiarize the students to academic skills required for a STEM student and demonstrate the role of STEM in addressing scientific and environmental justice issues of the Native American tribes. The students were asked to review the data presented in a technical report produced by the Yurok Fisheries Department on the 2002 Fish Kill and answer questions describing the conclusions of the scientists. They also performed a water quality experiment that involved collecting water in the Klamath and returning to campus to test if nitrogen is a limiting factor in the growth of *Microcystis aeruginosa*, a cyanobacteria that causes toxic algal blooms in the watershed.

### Peer Mentoring

The learning community partnered with the campus’s peer mentoring program called Retention through Academic Mentoring Program (RAMP), which utilizes 1:1 peer mentoring to guide first-year students in their development of positive academic habits and study skills, introduce them to campus culture, inform them about university policies and procedures, direct them to campus and community resources and services, and provide support through their transition to becoming college freshmen. The RAMP peer mentors were assigned a mentee caseload comprised of students in a single section of the First Year Experience course, augmented with additional students not in a learning community to bring each mentor caseload up to ~25 students.

### First Year Experience Course

Each student in the learning community was also enrolled in a 1-unit First Year Experience course (FYE) arranged by major. The FYE courses were led by faculty from the academic departments of the program. They worked together to develop a syllabus that combined a welcome to the major department and academic discipline, information on how to succeed as a STEM student, and an introduction to the various services on campus available to support personal and academic needs. Individual instructors agreed to a common set of learning objectives, but had considerable freedom to develop their own version for students in their major. The size of FYE section was at most 20 students.

### Cohorted Students in Block Scheduled First-Year Courses

Klamath Connection students were assigned specific sections of required major and general education (GE) courses that fulfilled both first-year major and university degree requirements. For fall semester, these included General Botany lecture and lab (major required and lower division life science GE), Communications (oral communication GE), Critical Thinking in Social and Environmental Sustainability (critical thinking GE), the first-year seminar, and a math course of the appropriate level. For the spring semester the courses varied by major, but all included English of the appropriate level (written communication GE), Native American Studies (social science GE), the next appropriate math course, and a lower division major requirement. Some classes were “exclusive,” meaning only students in the learning community were enrolled in the class (e.g., critical thinking general education course), whereas other courses mixed learning community students with other students (e.g., General Botany). The students’ fall semester was completely block enrolled (14–17 units) but only partially block enrolled in the spring term, enabling the opportunity to select the rest of their spring courses in consultation with their academic advisors.

The curriculum of the block courses was modified in slight but important ways. All instructors of block-enrolled courses were asked to aim at least some content of their course toward topics relevant to the Klamath River or Basin. This was facilitated by linking the summer immersion Klamath River water quality experiment into the major required classes. In the Fall semester, data from the experiment were analyzed in the students’ math courses, the logic of the research design was discussed in the critical thinking course, and the biology of the algae was discussed in General Botany. In the Spring semester, components of this topic were raised again in Chemistry, Wildlife, and Native American Studies courses by articulating them with an absorption spectroscopy laboratory, wildlife conservation and social and environmental justice, respectively.

### Participants

The first three cohorts of the learning community were 63, 116, and 118 students respectively, admitted in fall 2015, 2016, and 2017. The first cohort was composed of students enrolled in one the campus’s four largest STEM majors: Biology, Environmental Science, Wildlife, or Zoology. Students majoring in Botany, Environmental Resource Engineering, or Fisheries were added to the subsequent cohorts. First-year students admitted to one of the included majors were invited to “opt in” via paper and electronic invitations followed by more personalized calls and emails from staff and faculty. The invitations emphasized the opportunity to connect to peers and faculty, with local hands-on learning, guaranteed course enrollment, and academic support. Participants planning to reside on campus were given the option to live in Klamath Connection themed housing; we had capacity for 65% of them do so, and overall, more than 95% lived on campus in their first year. To simplify block-scheduling of the first cohort, only students ready for college-level math were included in the program; thereafter all students admitted to their majors were invited to participate regardless of math preparedness. This opt-in approach and college-ready requirement for the first cohort necessitated choosing an appropriate reference groups of students to minimize self-selection bias in outcomes (described in the Data Analysis section, below).

## Methods

Our work tests the hypothesis that participation in a place-based learning community elevated students’ sense of social belonging, academic skills and attitudes, and academic performance in gateway courses, leading to improved student persistence and narrowed equity gaps. We use a quasi-experimental design to compare the first-year outcomes of three cohorts of learning community students relative to a reference group of students.

We used propensity score matching to identify the reference group. Students were matched with the MatchIt package in R (Ho, Imai, King, Stuart, & Whitworth, 2018; R Core Team, 2018), using two continuous variables (high school GPA and number of AP units completed) and 4 binary variables (whether or not a student self-reported as female, from an underrepresented group, first-generation, and whether the student was designated by university admissions as being “college ready” in math, meaning their first math course would be college algebra or higher). We aimed to achieve a 2:1 match (2 reference for each learning community participant) and set the caliper width to 0.2 (Austin, 2011b). Limits in availability of matching students yielded an eventual match of 1.88:1; see Results for details and demonstration of baseline equivalence. Attrition – the loss of students in a sample during the course of a study – can also introduce bias because even though the learning community and reference group may have similar characteristics after matching, differences in attrition may cause members of the learning community and comparison groups to diverge over time, inhibiting rigorous statistical comparisons. In this study, analyses were restricted to first-year outcomes as the attrition from the fall to spring semester was small and not strongly different between the learning community and the reference group (7% overall, 5% difference), falling within a “low expected bias” (What Works Clearinghouse, 2017).

Analyses involved multiple response variables, described in detail below, that are associated with an integrated model of student persistence (Cabrera et al., 1993; Kerby, 2015). For all analyses we used four binary variables as predictor variables: learning community vs. reference, and yes/no status for underrepresented group, first-generation, and low-income. A student could belong to more than one student group. Cohort year (2015, 2016, 2017) was included as an additional covariate in initial models but since it was not significant it was removed from subsequent analyses. We tested global models with all possible interactions, and when they were absent emphasized additive models and models with only the URG × Learning Community interaction, as closing race-ethnicity equity gaps was a primary emphasis of our work. Low-income status was defined by whether or not a student received a federal Pell grant. Though most students in the learning community (69%) were grouped in the same residence hall (living learning community), preliminary analyses indicated these students’ outcomes did not differ substantively from those in the learning community but living elsewhere, so this variable was not included in subsequent analyses.

We used the Skyfactor©-Mapworks survey instrument to assess the impact of our program on the sense of belonging and academic skills and behaviors of students. The platform summarizes students’ responses to dozens of questions on a Likert scale (1–7) into factors that the literature suggests are associated with student retention and success, including 10 factors of students’ self-reported sense of belonging and community, and 10 factors associated with academic skills and behaviors (Woosley & Jones, 2011). All first-year students at the institution were asked to take the survey in the middle of their first fall semester and again mid-spring. Response rates are generally high (~60% fall; ~30% spring). Scores on factors were normally distributed, scaled such that large values reflect more favorable responses (e.g., a high homesickness score means a student is not feeling very distressed by homesickness), and we used two MANOVAs (one for the 10 factors of sense of belonging and community, one for the 10 factors of academic skills and behaviors) with factor score as the response variable and the predictor variables as described above.

To assess the program’s affect on academic achievement, we used ANOVA on first-year grade-point-average (GPA) and units completed at the institution in the first year. In addition, we examined the final grades earned in five key foundation science courses known to have comparatively low success rates, large equity gaps, and large sample sizes: Introductory Botany, Introductory Chemistry, College Algebra, Pre-calculus, (a student’s math preparedness at admission governs which math course is taken), and Introductory Zoology. A student grade of A, B, C, or Credit was categorized as a “success,” while a D, F, or unauthorized withdrawal was considered a “non-success.” Students receiving an incomplete or withdrawing early were removed from this analysis. For descriptive purposes (see Results) we also report the proportion of students earning each grade.

To examine first-year persistence, we quantified which students were still enrolled at the institution in the fall semester of their second year (“institutional persistence”), and which of these retained students were still in a declared STEM major (“STEM persistence”). Both values are reported as the percentage of students persisting. We used seven general linear models with logit link to examine course success (5 courses) and both institutional and STEM persistence using the same predictor variables as above. All data were obtained from the institution’s Office of Institutional Effectiveness, all analyses were conducted in R (R Core Team, 2018) with α = 0.05. The effect sizes are reported as Hedges’ g (for continuous variables) or odds ratios (for binary responses; WWC, 2017). We interpret small effects as Hedges’ g value of 0.2–0.49 and odds ratios of 1.5–3.49, and large effects as Hedges’ g values >0.8 and odds ratios >6.7, with medium effect sizes in between these values (Chen, Cohen, & Chen, 2010). All data collection, management, and analysis was completed under approval of the campus’s Institutional Review Board (IRB #15–238).

## Results

A total of 297 students participated in the learning community in 2015–16, 2016–17, and 2017–18. Since 27 students declined to self-report one or more demographic variable, they were removed from further analysis, yielding data for 270 students included in analyses, which corresponded to 18.1% of incoming first-time STEM students over these three years (1489, from which 96 were removed due to missing variables). While some programs aimed at closing equity gaps focus explicitly on underrepresented students, the place-based learning, the demographics of participants in our opt-in program differed modestly from the full pool of first-year STEM students, mainly in the proportion of students that were college ready in math. After propensity score matching, the matched reference group provided a statistically equivalent baseline to the participants, with all Hedges’ g and Cox indices below thresholds for small effect sizes (Table 1; WWC, 2017).

**Table 1:** Student demographics of students in the Klamath Connection learning community, a comparative reference group, and all first-year STEM students

Variable	Learning Community (*n* = 270)	Reference Group (*n* = 508)	All STEM (*n* = 1393)	Reference vs. Learning Community Hedges’ g or Cox Index^a^
High School GPA	3.49 ± 0.41	3.51 ± 0.45	3.30 ± 0.46	0.047
AP Units	10.97 ± 13.56	11.53 ± 12.71	7.26 ± 11.40	0.047
% Female	61.9%	61.6%	55.9%	0.006
% First-generation	44.1%	45.7%	52.9%	0.039
% URG	38.9%	39.6%	48.0%	0.043
% College-ready math	85.9%	85.0%	65.5%	0.017

^a^ Students are from academic years 2015–16, 2016–17, and 2017–18, and the reference group was identified by propensity score matching (see Methods), Hedges’ g (for High School GPA and AP units) and Cox index values (other variables) are provided, demonstrating baseline equivalence between the learning community and reference group of students

**Fig. 1:**
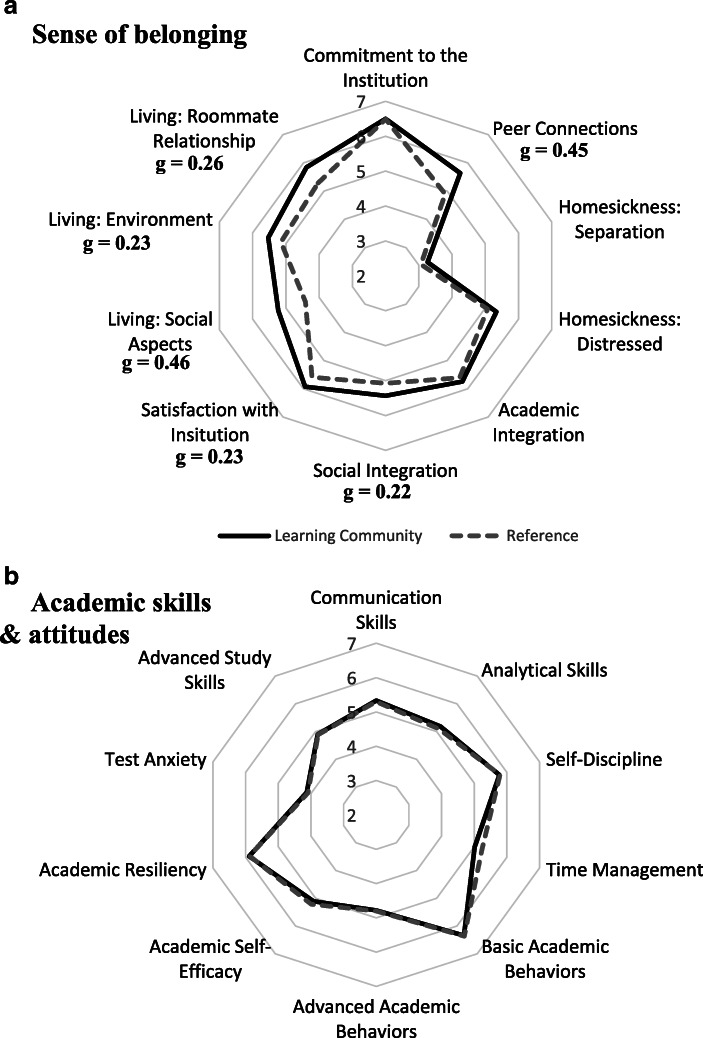
Students’ self-assessment of (**a**) sense of belonging and (**b**) academic skills and attitudes by students in the Klamath Connection learning community (*n* = 185) and in a reference set of students identified by propensity score matching (see Methods, *n* = 219). Scores are means of 20 factors pulled from the Mapworks (Skyfactor®) survey tool. Effect sizes were calculated as Hedges’ g and shown if ≥0.2

Surveys indicated that participating in the learning community prompted small increases in six of the ten factors of belonging and community (MANOVA *F*_10, 353_ = 3.98, *P* < 0.01; Fig. 1a, see Appendix Table 3 for full descriptive statistics). Stronger peer connections were self-reported by students in the learning community, and this was present for all students (Hedges’ g = 0.45), underrepresented students (g = 0.35), first-generation students (g = 0.55), and low-income students (g = 0.48). Likewise, learning community students in each of those groupings also reported higher satisfaction with the institution (g = 0.23–0.28) and better social aspects of on-campus living (g = 0.34–0.50). Improved on-campus living environment and better roommate relationships were reported by all students (g = 0.23 and 0.26), underrepresented students, (g = 0.24 and 0.40), and first-generation students (g = 0.27 and 0.26), but not by low-income students. A small effect of higher reported social integration was reported for all students overall (g = 0.22), but not for any of the student groups specifically. Of all ten belonging and community factors, the lowest scores were reported for homesickness, regardless of student group, and there was no strong effect of learning community participation on this factor (Fig. 1a). Survey response rates were 69% and 43% for the learning community and reference group, respectively.

The survey responses suggest the learning community did not substantively affect student perceptions of their academic skills and attitudes (MANOVA *F*_10, 420_ = 1.07, *P* = 0.38; Fig. 1b, see Appendix Table 3 for full descriptive statistics). Out of 40 possible effect sizes (4 student groups, and 10 factors), only three were above the 0.2 Hedges’ g threshold for a small effect. Underrepresented students and first-generation students in the learning community self-reported lower time management skills than did their counterparts in the reference group (g = 0.28 and 0.24, respectively), while low-income students and all students overall did not show this trend. Low-income students in the learning community also self-reported worse test anxiety (g = 0.22), a pattern that was not present for other student groups.

To examine academic achievement, we analyzed effects of the learning community on units completed at the institution in the first year, first year GPA, and course outcomes in key STEM courses for all students and disaggregated by underrepresented, first-generation, and low-income students (see Appendix Tables 4 and 5 for full descriptive statistics). There was a significant positive effect of learning community participation on units earned (*F*_1, 766_ = 19.17, *P* < 0.01). Students in the learning community completed 2.8–3.7 more units toward their degree in their first year than did students the reference group, corresponding to small effect sizes for all student groups (Table 2). The gap in average units earned between underrepresented students and their non-underrepresented counterparts shrank from −2.20 units in the reference group to less than one unit (−0.71) in the learning community, though this interaction was not statistically significant in an ANOVA. There were also small positive effect sizes of the learning community on first year GPAs, ranging from +0.09 to +0.27 grade points depending on student group (Table 2), though these effects were not statistically significant in an ANOVA (*F*_1, 766_ = 1.04, *P* = 0.31). The gap in GPA between underrepresented students and their non-underrepresented counterparts shrank from −0.33 grade points in the reference group to −0.12 in the learning community, though this did not correspond to significant interaction in an ANOVA.

**Table 2:** Comparison of units completed and grade point average (GPA) after the first year for students in the Klamath Connection learning community and in a reference set of students

	Learning Community		Reference		
Metric and student group	Mean ± S.D.	n	Mean ± S.D.	n	Hedges’ g
Units completed^a^					
All students	26.78 ± 7.82	270	23.99 ± 8.42	08	**0.34**
Underrepresented groups	26.34 ± 8.05	105	22.66 ± 8.64	01	**0.44**
First-generation	26.66 ± 8.09	119	23.19 ± 8.62	32	**0.27**
Low-income	26.97 ± 8.52	98	23.35 ± 8.38	21	**0.63**
First-year GPA					
All students	2.76 ± 0.92	270	2.67 ± 1.02	08	0.09
Underrepresented groups	2.69 ± 0.92	105	2.47 ± 1.04	01	**0.21**
First-generation	2.81 ± 0.94	119	2.54 ± 1.03	32	**0.27**
Low-income	2.74 ± 0.98	98	2.54 ± 1.02	21	0.19

Effect sizes are reported as Hedges’ g, with bolded values correspond to small (≥0.2) or medium (≥0.5) effect

^a^ Semester units completed at the institution in the first year; does not include AP units

**Fig. 2:**
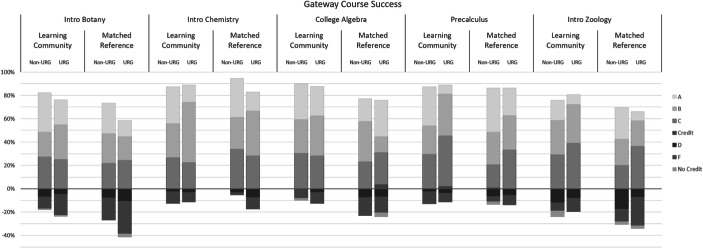
Grade outcomes for first-year STEM students in gateway foundational STEM courses, comparing students in the Klamath Connection place-based learning community, pooled over three academic years 2015–16 to 2017–18 (*n* = 270), to a reference set of first-time first-year STEM students identified by propensity score matching (see Methods, *n* = 494). Data are disaggregated by those self-reporting as belonging to an underrepresented groups (URG). The zero line distinguishes the % of students that succeeded in the course (depicted as positive values with the portion earning an A, B, C or Credit distinguished by shade) from those not succeeding (negative values, with the portion earning D, F or no credit distinguished by shading). Students withdrawing early from a class or receiving an incomplete were removed from analysis, but their inclusion had only modest effects on the values reported here, and these did not differ strongly between the learning community and the reference

Participation in the learning community led to higher passing rates in all of the five foundational STEM courses examined (Fig. 2). Effect sizes were small to medium for all student groups in Botany, Chemistry, Algebra, and Pre-calculus, and small for all but first generation and low-income students in Zoology, corresponding to increases in percent success ranging from +2.2% for Pre-calculus to +21.1% for College Algebra (Appendix Table 5). In addition, the gap in course pass rate between underrepresented students and their non-underrepresented counterparts shrank in all five courses, none were more pronounced than −6%, and in three courses (Zoology, Chemistry, Pre-calculus) the equity gap reversed (i.e., underrepresented students had higher success rates than non-underrepresented students; Fig. 2). Equity gaps in the percentage of students earning As and Bs were narrow, while gaps the percentage earning As did persist, though they were slightly reduced in PBLC participants (Appendix Table 5). The glm analysis revealed statistically significant main effects of learning community participation in botany (*z* = 2.90, *P* < 0.01) and college algebra (*z* = 2.46, *P* = 0.01), and a significant URG × Learning Community interaction for chemistry (*z* = 2.10, *P* = 0.04).

**Fig. 3:**
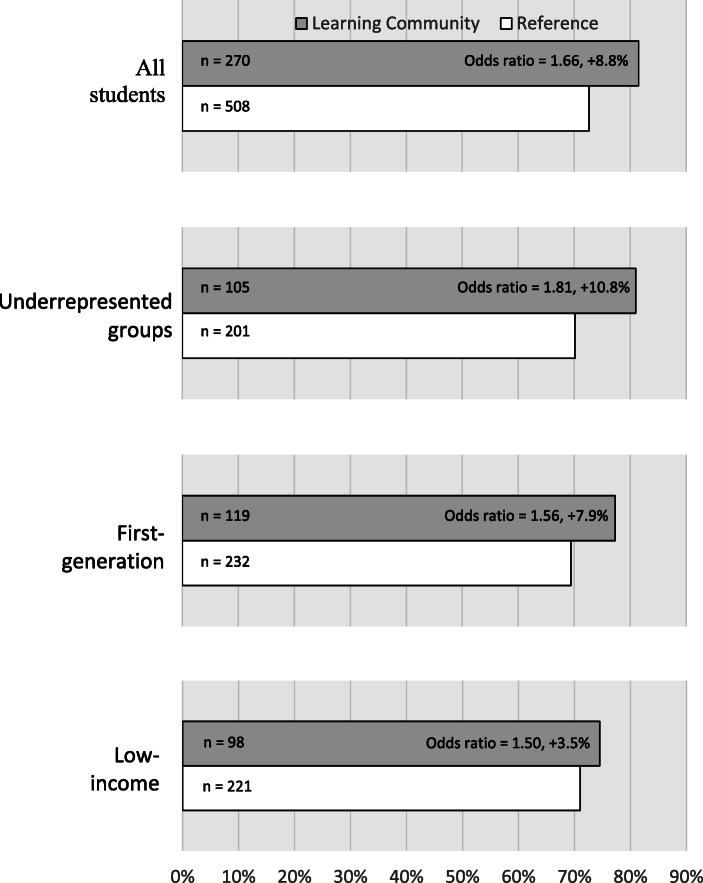
First-year persistence of first-time first-year STEM students at the institution, comparing students in the Klamath Connection place-based learning community, pooled over three academic years 2015–16 to 2017–18 (*n* = 270), to a reference set of first-time first-year STEM students identified by propensity score matching (see Methods, *n* = 508). Data are reported for all students, as well as those self-reporting as belonging to an underrepresented group, first-generation (first their family to attend college), and low-income. Effect size reported as odds ratios, and sample sizes are shown at the base of the bars

**Fig. 4:**
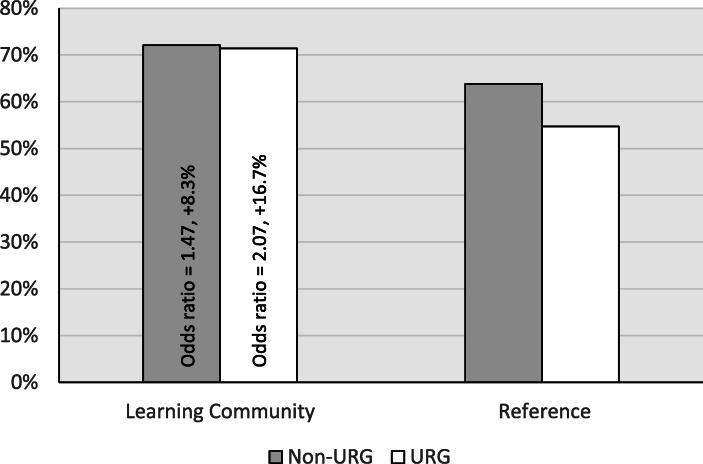
First-year retention of first-time first-year STEM students at the institution and remaining in a STEM major, comparing students in the Klamath Connection place-based learning community, pooled over three academic years 2015–16 to 2017–18 (n = 270), to a reference set of first-time first-year STEM students identified by propensity score matching (see Methods, n = 508). Data are disaggregated by whether or not students self-reported as belonging to an underrepresented groups (URG). Effect sizes reported as odds ratios

The learning community also raised rates and closed gaps in persistence. The general linear models showed a significant effect of learning community participation in first year institutional persistence (retained at the institution regardless of whether a student changed majors; *z* = 2.22, *P* = 0.03). Institutional persistence was 7.6 to 10.8 percentage points higher for students in the learning community, depending on student group, corresponding to a small effect size for underrepresented students specifically (Fig. 3). There was almost no gap in persistence between underrepresented groups and their non-underrepresented counterparts in the learning community (−0.9%) whereas it was −4.1% in the reference group. Persistence in a STEM major at the institution (STEM retention) was 8.3 to 16.7 percentage points higher for students in the learning community, depending on student group, corresponding to small effect sizes for all students, underrepresented students, and low-income students specifically, though there was no significant main effect of learning community in the glm of STEM persistence (*z* = 1.21, *P* = 0.23; see Appendix Table 4 for full descriptive statistics). The gap in STEM persistence between underrepresented students and their non-underrepresented counterparts shrank from −9.1% in the reference group to less than 1 % (−0.7%) in the learning community (Fig. 4).

## Discussion

This work provides the first evaluation of a place-based learning community designed to improve the academic outcomes of first-year STEM students. Using a quasi-experimental design, we found supportive evidence that linking the tenets of cohort-based learning communities and place-based education (Gruenewald & Smith, 2014; Semken & Freeman, 2008) can raise student outcomes and narrow equity gaps between underrepresented and non-underrepresented students in STEM. Overall, participants in the Klamath Connection had stronger sense of belonging (Fig. 1a), improved academic performance (Table 2, Fig. 2), and increased first-year persistence (Figs. 3 and 4) relative to a matched reference group. Equity gaps were narrowed in first year GPA, pass rates in several gateway STEM courses, and in first year persistence at the institution and in a STEM major specifically.

Our results suggest a place-based approach could be valuable for other institutions that attract large numbers of students of color to locations unlike that of their familial homes. By highlighting the contributions of Indigenous ways of knowing and emphasizing that a broad range of voices is needed to help solve complex social and environmental challenges, a place-based learning community can reinforce the well-documented social benefits of peer groups with a curriculum that explicitly values the role of individuality to the collective strength of an academic community. Underrepresented students participating in the learning community in this study, most of whom were not from nearby the institution, self-reported stronger sense of belonging and peer connections than non-participating underrepresented students (Fig. 1a), with no statistically identifiable gaps with their non-underrepresented counterparts. These results suggest that by highlighting the Native American communities of our region, Indigenous ways of knowing, and emphasizing that a broad range of voices is needed to help solve complex social and environmental challenges, a place-based learning community can reinforce the well-documented social benefits of peer groups with a curriculum that explicitly values the role of individuality to the collective strength of an academic community.

Watt and Badger’s (2009) belonging theory postulates that homesickness arises from one’s need to belong. They used this theory to study the effects of social belonging on homesickness, suggesting that because individuals tend to protect old social bonds in response to the need to belong, homesickness is actuated by the need to belong and generated in part from distress at the dissolution of former social bonds (Burt, 1993; Sun, Hagedorn, & Zhang, 2016). This work suggests that people whose belonging needs are not met in the new environment may surrender to feelings of distress (Brewin, Furnham, & Howes, 1989; Johnson et al., 2007; Strayhorn, 2008; Scharp, Paxman, & Thomas, 2016). Coupling this theoretical lens with the empirical evidence reported here for the positive impact of social connection for learning community participants, we expected that students’ self-reported homesickness would have been eased for participants. We did not find strong evidence for this, however. Scores for navigating homesickness were only marginally higher for learning community participants than non-participants (Fig. 1a), and the gap between underrepresented and non-underrepresented students was similar for participant and non-participants. Previous research has distinguished “homesickness distress”– corresponding to feelings of regret for leaving and isolation – and “homesickness separation”– corresponding to missing friends and family from back home and thinking about them often (Paul & Brier, 2001; Thurber & Walton, 2012). Our results showed that students self-reported significantly lower feeling of homesickness distress than separation, though these measures did not differ substantively between participants and non-participants. Additional work is needed to better understand how the academic welcoming and sense of belonging fostered by a learning community can help students cope with homesickness.

It is vital to recognize that a sense of place arises not just from a location, but from interrelationships between people and place (Kerby, 2015; Kimmerer, 2013; Pretty et al., 2003). Moreover, a socially just placed-based education must respect the epistemological traditions of a place and its Indigenous community (Seawright, 2014). For the learning community in this study, which focused on life sciences, these recognitions prompted curricular changes to give better credence to other ways of knowing alongside Western scientific epistemologies, especially Traditional Ecological Knowledge in introductory courses (McKinley & Gan, 2014), a process that is ongoing at the institution (Sprowles et al., 2019), with newly launching place-based learning communities also including a first-year Native American studies course in the blocked schedule and linked content with STEM courses. The damaging colonial history of STEM and higher education (Aikenhead & Elliott, 2010), the improvement of science by broadening voices and perspectives (Page, 2008; Gibbs, 2014), and the relevance of issues faced by local Indigenous communities to the experiences of other minoritized students and their families (Alkholy, Gendron, McKenna, Dahms, & Ferreira, 2017) all converge to demand that faculty work to honor different ways of knowing and integrate Indigenous epistemologies into first-year curricula. While we cannot yet claim much experience or elegance in integrating these concepts, we assert that any place-based education must strive for that goal.

Though the results reported here provide support for place-based learning communities as an important mechanism for STEM reform and improved academic outcomes, Klamath Connection participants did not self-report significant gains in academic skills and behaviors over those reported by individuals in the matched reference group. (Fig. 1b). Furthermore, equity gaps persisted in the percentage of As earned by underrepresented students when compared to their top-performing non-URM counterparts (Appendix Table 5). We predicted that by integrating the scientific, environmental and civic concerns of the Native American tribes of the Klamath Basin into the STEM curriculum of Klamath Connection we would see gains in academic excellence of first year STEM students from minoritized backgrounds. We are analyzing Klamath Connection participant coursework and survey data to better understand the effectiveness of the programming on cultivating awareness and understanding of social and environmental justice issues experienced by Native people, gains in intercultural knowledge, and the importance of cross-cultural communication skills for STEM professionals (Sprowles et al., in prep).

There are several possible explanations for no measurable improvement of academic behaviors. The social benefits and sense of belonging may have been sufficient to prompt the observed improvements in academic performance and retention, as described by Hurtado and Carter (1997) and Hausmann, Schofield, and Woods (2007). Alternatively, no impact on academic behaviors may have been an artefact of our study design. The Mapworks© survey instrument was administered in weeks 4–7 of the first semester. This instrument may have been unable to accurately assess students’ academic skills either because it relies on student self-assessment, which may be insensitive to short-term improvement (Bowman, 2010), or because of insufficient time elapsed for the programming to have yielded perceived improvement (Duckworth & Yeager, 2015). Furthermore, the attention to time management, study practices, and pre- and post-exam reflections in the First-Year Experience course and work with peer mentors may have accelerated the students’ recognition of the demands of university-level study, or possibly elevated anxiety, which impacted their self-assessment of academic behaviors. In contrast, the responses of students in the reference group, most of whom had not yet engaged much with classroom attention to time budgets and study habits by the time of the survey, may reflect a level of naiveté in the early fall semester (Kruger & Dunning, 1999). A possible selection bias, whereby participants with academic anxiety were attracted to the program by its academic supports, may also be operating. Continued monitoring of these three opt-in cohorts and newer cohorts that are “opt-out, should enable us to evaluate these and distinguish among these non-mutually exclusive possible explanations. Future work should also examine possible benefits of place-based learning communities on integrated and field-based learning.

Another important caveat of the results reported here is that the effect sizes were in some cases smaller for low-income students. For example, participation in the learning community increased first-year persistence by 10.8% and 7.9% for underrepresented and first-generation students, respectively, but it only increased by 3.5% for low-income students (Fig. 3). This likely reflects the reality that while the place-based learning community substantively improved the sense of belonging and provided curricular relevance to low-income students, it was unable to address extra-curricular financial challenges (e.g. tuition, housing, food, healthcare) faced by low-income students.

In summary, the place-based learning community examined here demonstrably improved students’ sense of belonging, academic performance, and first-year persistence, and it narrowed equity gaps in multiple measures. While the design of the Klamath Connection was necessarily tailored to this institution, the deliberate attention to linking a sense of place to an interdisciplinary curriculum that transforms the first year experience may have general application to many other institutions, perhaps especially those that attract students to places unlike that of that their familial homes.

## Figures and Tables

**Table 3:**
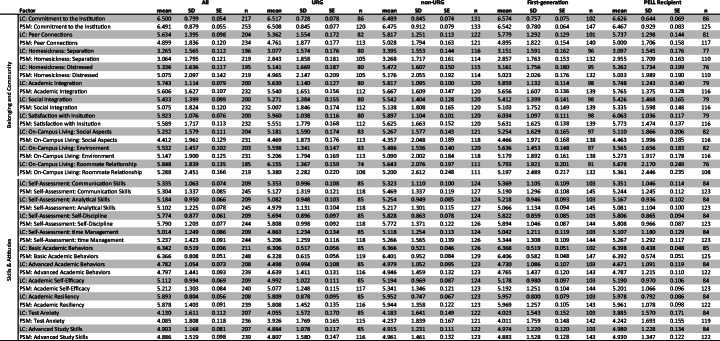
Students’ self-reported sense of belonging, community, academic skills and behaviors, as indicated by responses to Skyfactor Mapworks surveys

Results are provided for 20 factors each of which is comprised of 2–4 questions on an ordinal likert-scale of 1 to 7. Larger value indicator more favorable responses (i.e., high scores indicate strong sense of belonging, low feelings of homesickness, etc.). Means ±1 S.D. for all students and disaggregated student groups are provided, both for students in the learning community (LC; gray rows) and in the propensity score matched reference group (PSM; white rows)

**Table 4:**
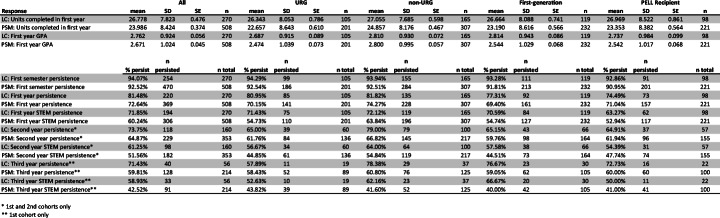
Academic achievement metrics obtained from students’ academic records

Means ±1 S.D. for all students and disaggregated student groups are provided, both for students in the learning community (LC; gray rows) and in the propensity score matched reference group (PSM; white rows)

**Table 5:**
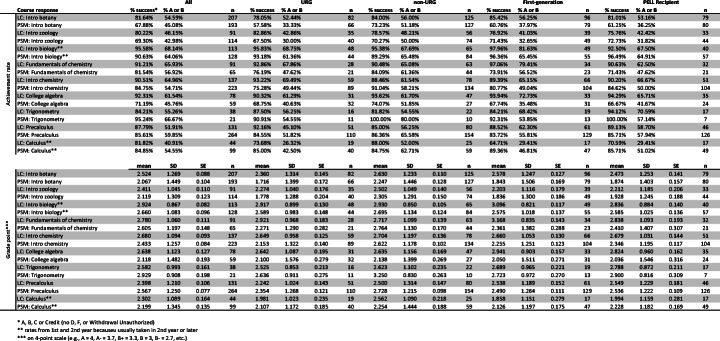
Student achievement in gateway STEM courses obtained from students’ academic records

Results for all students and disaggregated student groups are provided, both for students in the learning community (LC; gray rows) and in the propensity score matched reference group (PSM; white rows). The Following Tables have grades of I and W removed from calculations

## Data Availability

All data are available from the lead author upon request. Analysis code is available from Steven Margell.

## References

[CR1] Aikenhead, G. S., & Elliott, D. (2010). An emerging decolonizing science education in Canada. *Canadian Journal of Science, Mathematics and Technology Education*, *10*(4), 321–338.

[CR2] Alkholy, S. O., Gendron, F., McKenna, B., Dahms, T., & Ferreira, M. P. (2017). Convergence of Indigenous Science and western Science Impacts student’s Interest in STEM and Identity as a Scientist. *Ubiquitous Learning: An International Journal**10*(1): 1–13.

[CR3] American Chemical Society. National meeting. (2010). *Science education and civic engagement: The SENCER approach*. American Chemical Society.

[CR4] Asai, D. (2016). A new strategy to build capacity for creativity. Retrieved from https://www.hhmi.org/content/new-strategy-build-capacity-creativity-science-education. Accessed July 2018.

[CR6] Austin, P. C. (2011a). An introduction to propensity score methods for reducing the effects of confounding in observational studies. *Multivariate Behavioral Research*, *46*(3), 399–424. 10.1080/00273171.2011.568786 PMC3144483 21818162

[CR7] Austin, P. C. (2011b). Optimal caliper widths for propensity-score matching when estimating differences in means and differences in proportions in observational studies. *Pharmaceutical Statistics*, *10*(2), 150–161. 10.1002/pst.433 PMC3120982 20925139

[CR8] Bean, J. P. (1980). Dropouts and turnover: The synthesis and test of a causal model of student attrition. *Research in Higher Education*, *12*(2), 155–187.

[CR9] Bean, J. P., & Metzner, B. S. (1985). A conceptual model of non-traditional undergraduate student attrition. *Review of Educational Research*, *55*(4), 485–540.

[CR10] Bowman, N. A. (2010). Can 1st-year college students accurately report their learning and development?. *American Educational Research Journal*, *47*(2), 466–496.

[CR11] Brewin, C. R., Furnham, A., & Howes, M. (1989). Demographic and psychological determinants of homesickness and confiding among students. *British Journal of Psychology*, *80*(4), 467–477.

[CR12] Brown, B. (2017). *Braving the wilderness: The quest for true belonging and the courage to stand alone*. New York, New York, USA: Random House.

[CR13] Brownell, J. E., & Swaner, L. E. (2009). High-impact practices: Applying the learning outcomes literature to the development of successful campus programs. *Peer Review*, *11*(2), 26.

[CR14] Burgette, J. E., & Magun-Jackson, S. (2008). Freshman orientation, persistence, and achievement: A longitudinal analysis. *Journal of College Student Retention: Research, Theory & Practice*, *10*(3), 235–263.

[CR15] Burt, C. D. (1993). Concentration and academic ability following transition to university: An investigation of the effects of homesickness. *Journal of Environmental Psychology*, *13*(4), 333–342.

[CR16] Cabrera, A. F., Nora, A., & Castaneda, M. B. (1993). College persistence: Structural equations modeling test of an integrated model of student retention. *The Journal of Higher Education*, *64*(2), 123–139.

[CR17] Cabrera, A. F., Nora, A., Terenzini, P. T., Pascarella, E., & Hagedorn, L. S. (1999). Campus racial climate and the adjustment of students to college: A comparison between White students and African-American students. *The Journal of Higher Education*, *70*(2), 134–160.

[CR18] Carrino, S.S., Gerace, W.J. (2016). Why STEM learning communities work: The development of physoscocial learning factors through social interaction*. Learning Communities Research and Practice*, *4*(1), Article 3.

[CR19] Chen, H., Cohen, P., & Chen, S. (2010). How big is a big odds ratio? Interpreting the magnitudes of odds ratios in epidemiological studies. *Communications in Statistics—Simulation and Computation®*, *39*(4), 860–864.

[CR20] Clark, M. R. (2005). Negotiating the freshman year: Challenges and strategies among first-year college students. *Journal of College Student Development*, *46*(3), 296–316.

[CR21] Dagley, M., Georgiopoulos, M., Reece, A., & Young, C. (2016). Increasing retention and graduation rates through a STEM learning community*. Journal of College Student Retention: Research Theory & Practice,* 18(2), 167–182.

[CR22] Davidson, C., & Wilson, K. (2013). Reassessing Tinto's concepts of social and academic integration in student retention. *Journal of College Student Retention: Research, Theory & Practice*, *15*(3), 329–346.

[CR23] de Brey, C., Musu, L., McFarland, J., Wilkinson-Flicker, S., Diliberti, M., Zhang, A., Branstetter, C., and Wang, X. (2019). Status and Trends in the Education of Racial and Ethnic Groups 2018 (NCES 2019-038). U.S. Department of Education. Washington, DC: National Center for Education Statistics. Retrieved from https://nces.ed.gov/pubsearch/. Accessed Sep 2019.

[CR24] Duckworth, A. L., & Yeager, D. S. (2015). Measurement matters: Assessing personal qualities other than cognitive ability for educational purposes. *Educational Researcher*, *44*(4), 237–251. 10.3102/0013189X15584327 PMC4849415 27134288

[CR25] Estrada, M., Burnett, M., Campbell, A. G., Campbell, P. B., Denetclaw, W. F., Gutiérrez, C. G., ... & Okpodu, C. M. (2016). Improving underrepresented minority student persistence in STEM. *CBE—Life Sciences Education*, *15*(3), es5. 10.1187/cbe.16-01-0038 PMC5008901 27543633

[CR26] Gándara, P (1999). Priming the Pump: Strategies for Increasing the Achievement of Underrepresented Minority Undergraduates. Princeton, N.J.: College Board.

[CR27] Garcia, G. A. (2019). *Becoming Hispanic-serving institutions: Opportunities for colleges and universities*. Charles Village, Baltimore, USA: Johns Hopkins University Press.

[CR28] Gibbs, K. (2014). Diversity in STEM: What it is and why it matters. *Scientific American, 10*. Retrieved from: https://blogs.scientificamerican.com/voices/diversity-in-stem-what-it-is-and-why-it-matters/. Accessed July 2018.

[CR29] Gillespie, P. & Petitubin, M. (2016) Stem learning communities. *Journal of Education & Social Policy*, *3*(3), 1–6.

[CR30] Goldberg, B., & Finkelstein, M. (2002). Effects of a first-semester learning community on non-traditional technical students. *Innovative Higher Education*, *26*(4), 235–249.

[CR31] González, K. P. (2002). Campus culture and the experiences of Chicano students in a predominantly White university. *Urban Education*, *37*(2), 193–218.

[CR32] Gordon, E. (1986). *A descriptive analysis of programs and trends in engineering education for ethnic minority students. A report to the field*. New Haven: Institution for Social and Policy Studies, Yale University.

[CR33] Graham, M. J., Frederick, J., Byars-Winston, A., Hunter, A. B., & Handelsman, J. (2013). Increasing persistence of college students in STEM. *Science*, *341*(6153), 1455–1456. 10.1126/science.1240487 PMC10167736 24072909

[CR34] Gruenewald, D. A., & Smith, G. A. (Eds.). (2014). *Place-based education in the global age: Local diversity*. Milton Park, Abingdon, Oxfordshire, UK: Routledge.

[CR35] Guiffrida, D. A. (2006). Toward a cultural advancement of Tinto's theory. *The Review of Higher Education*, *29*(4), 451–472.

[CR36] Hale, F. W. (2004). *What makes racial diversity work in higher education: Academic leaders present successful policies and strategies*. Sterling, Virginia, USA: Stylus Publishing, LLC.

[CR37] Hausmann, L. R., Schofield, J. W., & Woods, R. L. (2007). Sense of belonging as a predictor of intentions to persist among African American and White first-year college students. *Research in Higher Education*, *48*(7), 803–839.

[CR38] Ho, D., K. Imai, G. King, E. Stuart, & A. Whitworth. (2018). *Package ‘MatchIt’: Nonparametric preprocessing for parametric causal inference*. Retrieved from: http://gking.harvard.edu/matchit. Accessed July 2018.

[CR39] Holton, M. (2015). ‘I already know the city, I don't have to explore it’: Adjustments to ‘sense of place’for ‘local’UK university students. *Population, Space and Place*, *21*(8), 820–831.

[CR40] Hurtado, S., Cabrera, N. L., Lin, M. H., Arellano, L., & Espinosa, L. L. (2009). Diversifying science: Underrepresented student experiences in structured research programs. *Research in Higher Education*, *50*(2), 189–214. 10.1007/s11162-008-9114-7 PMC3596157 23503690

[CR41] Hurtado, S., & Carter, D. F. (1997). Effects of college transition and perceptions of the campus racial climate on Latino college students' sense of belonging. *Sociology of education 70*, 324–345.

[CR42] Hurtado, S., Eagan, M. K., Cabrera, N. L., Lin, M. H., Park, J., & Lopez, M. (2008). Training future scientists: Predicting first-year minority student participation in health science research. *Research in Higher Education*, *49*(2), 126–152. 10.1007/s11162-007-9068-1 PMC3596162 23503996

[CR43] Johnson, D. R., Soldner, M., Leonard, J. B., Alvarez, P., Inkelas, K. K., Rowan-Kenyon, H. T., & Longerbeam, S. D. (2007). Examining sense of belonging among first-year undergraduates from different racial/ethnic groups. *Journal of College Student Development*, *48*(5), 525–542.

[CR44] Johnson, M., Sprowles, A., Overeem, K., & Rich, A. (2017). A Place-based learning community: Klamath Connection at Humboldt State University. *Learning Communities Research and Practice*, *5*(2), 4.

[CR45] Kerby, M. B. (2015). Toward a new predictive model of student retention in higher education: An application of classical sociological theory. *Journal of College Student Retention: Research, Theory & Practice*, *17*(2), 138–161.

[CR46] Kimmerer, R. W. (2013). *Braiding sweetgrass: Indigenous wisdom, scientific knowledge and the teachings of plants*. Milkweed Editions. Minneapolis, Minnesota, USA.

[CR47] Klein, J. T. (2005). Integrative learning and interdisciplinary studies. *Peer Review*, *7*(4), 8–10.

[CR48] Knapp, C. E. (2005). The “I–thou” relationship, place-based education, and Aldo Leopold. The *Journal of Experimental Education*, *27*(3), 277–285.

[CR49] Kruger, J., & Dunning, D. (1999). Unskilled and unaware of it: How difficulties in recognizing one's own incompetence lead to inflated self-assessments. *Journal of Personality and Social Psychology*, *77*(6), 1121. 10.1037//0022-3514.77.6.1121 10626367

[CR50] Kuh, G. D. (2008). *High-Impact educational practices: What they are. Who has access to them, and why they matter*. American Association of Colleges and Universities.

[CR51] Mack, K. M., Winter, K., & Soto, M. (Eds.). (2019). *Culturally responsive strategies for reforming STEM higher education: Turning the TIDES on inequity*. Bingley, UK: Emerald Publishing Limited.

[CR52] McGee, E., & Bentley, L. (2017). The equity ethic: Black and Latinx college students reengineering their STEM careers toward justice. *American Journal of Education*, *124*(1), 1–36.

[CR53] McKinley, E., & Gan, M. J. (2014). Culturally responsive science education for indigenous and ethnic minority students. *Handbook of Research on Science Education*, *2*, 284–300.

[CR54] Medina, J. (2013). *The epistemology of resistance: Gender and racial oppression, epistemic injustice, and the social imagination*. Oxford, UK: Oxford University Press.

[CR55] Museus, S. D. (2014). The culturally engaging campus environments (CECE) model: A new theory of success among racially diverse college student populations. In *Higher education: Handbook of theory and research* (pp. 189–227). Springer, Dordrecht.

[CR56] National Academy of Sciences, National Academy of Engineering, and Institute of Medicine. (2011) *Expanding underrepresented minority participation: America's Science and Technology Talent at the Crossroads*. Washington, DC: The National Academies Press. 22379652

[CR57] National Leadership Council for Liberal Education and America's Promise. (2007). *College learning for the new global century*. American Association of Colleges and Universities.

[CR58] National Science Foundation, National Center for Science and Engineering Statistics. (2015). *Characteristics of scientists and engineers in the United States*: 2013. http://ncsesdata.nsf.gov/us-workforce/2013/. Accessed Sep 2019.

[CR59] O'Keeffe, P. (2013). A sense of belonging: Improving student retention. *College Student Journal*, *47*(4), 605–613.

[CR60] Olson, S., & Riordan, D. G. (2012). Engage to Excel: Producing One Million Additional College Graduates with Degrees in Science, Technology, Engineering, and Mathematics. Report to the President. *Executive Office of the President*.

[CR61] Orr, D. W. (2004). *Earth in mind: On education, environment, and the human prospect*. Washington, D.C., USA: Island Press.

[CR62] Otto, S., Evins, M. A., Boyer-Pennington, M., & Brinthaupt, T. M. (2015). Learning communities in higher education: Best practices. *Journal of Student Success and Retention*, 2(1), 1–20.

[CR63] Page, S. E. (2008). *The difference: How the power of diversity creates better groups, firms, schools, and societies-new edition*. Princeton, New Jersey, USA: Princeton University Press.

[CR65] Paul, E. L., & Brier, S. (2001). Friendsickness in the transition to college: Precollege predictors and college adjustment correlates. *Journal of Counseling & Development*, *79*(1), 77–89.

[CR66] Pretty, G. H., Chipuer, H. M., & Bramston, P. (2003). Sense of place amongst adolescents and adults in two rural Australian towns: The discriminating features of place attachment, sense of community and place dependence in relation to place identity. *Journal of Environmental Psychology*, *23*(3), 273–287.

[CR67] R Core Team (2018) R: A language and environment for statistical computing. *R Foundation for Statistical Computing, Vienna*. https://www.R-project.org. Accessed July 2018.

[CR68] Rendón, L. I. (1994). Validating culturally diverse students: Toward a new model of learning and student development. *Innovative Higher Education*, *19*(1), 33–51.

[CR69] Rendón, L. I., Garcia, M., & Person, D. (2004). *Transforming the first year of college for students of color. The first-year experience monograph series no. 38*. National Resource Center for The First-Year Experience and Students in Transition. University of South Carolina, 1728 College Street, Columbia, SC 29208.

[CR70] Riggs, B. (2018). Mutually beneficial research partnerships for equity and innovation in science. *American Society of Cell Biology*. Retrieved from: https://www.ascb.org/careers/mutually-beneficial-research-partnerships-for-equity-and-innovation-in-science/. Accessed July 2018.

[CR71] Scharp, K. M., Paxman, C. G., & Thomas, L. J. (2016). “I want to go home” homesickness experiences and social-support-seeking practices. *Environment and Behavior*, *48*(9), 1175–1197.

[CR72] Seawright, G. (2014). Settler traditions of place: Making explicit the epistemological legacy of white supremacy and settler colonialism for place-based education. *Educational Studies*, *50*(6), 554–572.

[CR73] Semken, S., & Freeman, C. B. (2008). Sense of place in the practice and assessment of place-based science teaching. *Science Education*, *92*(6), 1042–1057.

[CR74] Shapiro, D., Dundar, A., Huie, F., Wakhungu, P. K., Yuan, X., Nathan, A., & Hwang, Y. (2017). A national view of student attainment rates by race and ethnicity–Fall 2010 cohort. (Signature Report No. 12b). Herndon, VA: National Student Clearinghouse Research Center.

[CR75] Smith, B. L., MacGregor, J., Matthews, R., & Gabelnick, F. (2009). *Learning communities: Reforming undergraduate education*. Hoboken, New Jersey, USA: Wiley Publishing.

[CR76] Smith, G. A. (2002). Place-based education: Learning to be where we are. *Phi Delta Kappan*, *83*(8), 584–594.

[CR77] Solanki, S., McPartlan, P., Xu, D. and Sato, B.K., (2019). Success with EASE: Who benefits from a STEM learning community? *PLoS One*, *14*(3), 1–20. 10.1371/journal.pone.0213827 PMC6430422 30901339

[CR78] Sommo, C., Mayer K., Rudd, T., & D. Cullinan. (2012). *Opening doors: Commencement day: Six year effects of a freshman learning community program at Kingsborough Community College./Sommo Colleen*. MDRC.

[CR79] Sprowles, A., et al. (2019). Place-based learning communities on a rural campus: Turning challenges into assets. *Learning Communities Research and Practice 7*,(1), 6–16.

[CR80] Sprowles, A.E., M.D. Johnson, L. Hillman, K. Malloy, K. Goldenberg, & S. Margell. (In prep.) Assessing the impact of integrating the science, culture, values, and social-justice concerns of contemporary Native American people on the academic achievement of students from racially minoritized groups in a first-year STEM learning community.

[CR81] Stassen, M. L. (2003). Student outcomes: The impact of varying living-learning community models. *Research in Higher Education*, *44*(5), 581–613.

[CR82] Strayhorn, T. L. (2008). Fittin' in: Do diverse interactions with peers affect sense of belonging for Black men at predominantly White institutions? *NASPA Journal*, *45*(4), 501–527.

[CR83] Strayhorn, T. L. (2018). *College students' sense of belonging: A key to educational success for all students*. Milton Park, Abingdon, Oxfordshire, UK: Routledge.

[CR84] Sun, J., Hagedorn, L. S., & Y. Zhang (2016). Homesickness at college: Its impact on academic performance and retention. *Journal of College Student Development*, *57*(8), 943–957.

[CR85] Thoman, D.B., Brown, E. R., Mason, A. Z., Harmsen, A.G., and Smith, J.L. (2014). The role of altruistic values in motivating underrepresented minority students for biomedicine. *BioScience**65**(*2*)*, 183–188. 10.1093/biosci/biu199 PMC4731875 26834259

[CR86] Thurber, C. A., & Walton, E. A. (2012). Homesickness and adjustment in university students. *Journal of American College Health*, *60*(5), 415–419. 10.1080/07448481.2012.673520 22686364

[CR87] Tinto, V. (1975). Dropout from higher education: A theoretical synthesis of recent research. *Review of Educational Research*, *45*(1), 89–125.

[CR88] Tinto, V. (2003). Learning better together: The impact of learning communities on student success. *Higher Education Monograph Series*, *1*(8), 1–8.

[CR89] U.S. Census Bureau QuickFacts selected: Arcata city, California. (2010). Retrieved from https://www.census.gov/quickfacts/fact/dashboard/arcatacitycalifornia/AGE765210. Accessed July 2018.

[CR90] Watt, S. E., & Badger, A. J. (2009). Effects of social belonging on homesickness: An application of the belongingness hypothesis. *Personality and Social Psychology Bulletin*, *35*(4), 516–530. 10.1177/0146167208329695 19193602

[CR91] Weiss, M. J., Visher, M. G., Weissman, E., & Wathington, H. (2015). The impact of learning communities for students in developmental education: A synthesis of findings from randomized trials at six community colleges. *Educational Evaluation and Policy Analysis*, *37*(4), 520–541.

[CR92] What Works Clearinghouse. 2017. Procedures Handbook, Version 4. https://ies.ed.gov/ncee/wwc/Docs/referenceresources/wwc_standards_handbook_v4.pdf. Accessed July 2018.

[CR93] Wilmer, E. (2009). The influence of learning communities on the interaction levels of developmental english students. *Inquiry*, *14*(1), 55–67.

[CR94] Woodhouse, J. L., & Knapp, C. E. (2000). Place-based curriculum and instruction: Outdoor and environmental dducation approaches. *ERIC Digest*.

[CR95] Woosley, S. & D. Jones (2011). *The Foundation of MAP-Works*. Retrieved from: http://www2.indstate.edu/studentsuccess/pdf/The%20Foundation%20of%20MAP-Works.pdf. Accessed July 2018.

[CR96] Zhao, C. M., & Kuh, G. D. (2004). Adding value: Learning communities and student engagement. *Research in Higher Education*, *45*(2), 115–138.

